# Vitamin D Supplementation and Vitamin D Status during Pregnancy and the Risk of Congenital Anomalies—A Systematic Review and Meta-Analysis

**DOI:** 10.3390/nu15092125

**Published:** 2023-04-28

**Authors:** Karen Christina Walker, Fanney Thorsteinsdottir, Henrik Thybo Christesen, Vibeke Elisabeth Hjortdal, Berit Lilienthal Heitmann, Ina Olmer Specht, Mina Nicole Händel

**Affiliations:** 1 Research Unit for Dietary Studies at The Parker Institute, Bispebjerg and Frederiksberg Hospital, Part of the Copenhagen University Hospital, The Capital Region, Nordre Fasanvej 57, Vej 8, Indgang 11, 2000 Frederiksberg, Denmark; 2 Hans Christian Andersen Children’s Hospital, Odense University Hospital, 5000 Odense, Denmark; 3 Department of Clinical Research, University of Southern Denmark, 5230 Odense, Denmark; 4 Department Cardiothoracic Surgery, Rigshospitalet, Copenhagen University Hospital, 2100 Copenhagen, Denmark; 5 The Boden Initiative, The Charles Perkins Centre, The University of Sydney, Sydney, NSW 2006, Australia; 6 The Research Unit for General Practice and Section of General Practice, Department of Public Health, University of Copenhagen, 1353 Copenhagen, Denmark; 7 Research Unit OPEN, Department of Clinical Research, University of Southern Denmark, 5230 Odense, Denmark

**Keywords:** prenatal exposure, vitamin D, congenital anomalies, systematic review

## Abstract

Maternal dietary factors have been suggested as possible contributing influences for congenital anomalies (CAs). We aimed to assess the association between vitamin D supplementation or vitamin D status (s-25OHD) during pregnancy and CAs in the offspring. A comprehensive literature search was conducted in the three electronic databases: PubMed, Embase, and Cochrane Library. Included studies were critically appraised using appropriate tools (risk of bias 2, ROBINS-I). A protocol was registered in the International Prospective Register of Systematic Reviews (CRD42019127131). A meta-analysis of four randomised controlled trials (RCTs) including 3931 participants showed no effect of vitamin D supplementation on CAs, a relative risk of 0.76 (95% CI 0.45; 1.30), with moderate certainty in the effect estimates by GRADE assessment. Of the nine identified observational studies, six were excluded due to a critical risk of bias in accordance with ROBINS-I. Among the included observational studies, two studies found no association, whereas one case-control study identified an association between s-25OHD < 20 nmol/L and neural tube defects, with an adjusted odds ratio of 2.34 (95% CI: 1.07; 5.07). Interpretation of the results should be cautious given the low prevalence of CAs, RCTs with onset of supplementation after organogenesis, and low-quality observational studies.

## 1. Introduction

Structural congenital anomalies (CAs) occur during intrauterine life, may present in different organ systems, and vary in complexity. Globally, an estimated 6% of infants are born with CAs, and the most common forms of CAs are congenital heart disease (CHD) or neural tube defects (NTDs) [1,2]. CAs are a major cause of infant morbidity and long-term disability and are a leading cause of infant mortality globally. Known causes include gene defects and chromosomal abnormalities, including trisomy 13 and 18. Still, many CAs occur without a known cause [2]. Several environmental and potentially modifiable factors have been suggested as potential risk factors for CAs, including maternal illnesses, drugs, pollution, and maternal malnutrition [1]. For instance, a case-control study from 2016 found a reduced occurrence of selected CHDs in the offspring with increased maternal diet quality [3]. Early folic acid supplementation has specifically been shown to protect against NTDs and thus is advised to women planning pregnancy [4]. Additionally, a Dutch case-control study from 2018 found that a compromised maternal vitamin D status (serum 25-hydroxyvitamin D; s-25OHD) was associated with a higher prevalence of CHD in offspring [5]. However, this study used post-pregnancy s-25OHD concentrations as a proxy for exposure during early pregnancy [5]. s-25OHD is the primary circulating form of vitamin D and is used to determine vitamin D status. The s-25OHD concentrations do not differ between pregnant and non-pregnant women, whereas the active form of vitamin D (1,25(OH)_2_D) increases substantially in pregnancy [6]. During pregnancy, the supply of vitamin D in the foetus completely depends on maternal concentrations of vitamin D, and concentrations in the neonate, cord, and mother are closely correlated [7,8]. 

A critical window for intervention in relation to the prevention of CAs might be in early pregnancy when the foetal organ systems start developing [9]. The objective of the present systematic review and meta-analysis was to synthesise and critically appraise the literature for studies that examined the association between exposure to vitamin D status during pregnancy and the risk of CAs in the offspring. 

## 2. Methods

Before conducting the present systematic review, we developed a protocol based on Preferred Reporting Items for Systematic Review and Meta-analysis Protocols (PRISMA-P) [10], which we registered at the International Prospective Register of Systematic Reviews (PROSPERO) (registration number: CRD42019127131, 14 May 2019). The present systematic review was reported according to the Preferred Reporting Items for Systematic Reviews and Meta-analyses (PRISMA) statement [11]. 

### 2.1. Alterations to Protocol

Quality assessment of randomised controlled trials (RCTs) was performed using Cochrane Risk of Bias 2 (RoB 2) [12] and not Cochrane Risk of Bias (RoB) [13] as stated in the protocol. We found the updated tool the better choice. 

### 2.2. Eligibility Criteria

Studies in English on the association between vitamin D exposure during foetal life and the development of CAs in offspring were eligible for inclusion. The eligibility criteria were structured as Population, Intervention (exposure), Comparison, and Outcome (PICO). The population of interest was healthy pregnant women without any disease prior to pregnancy (e.g., diabetes mellitus) or any diseases developed in pregnancy (e.g., gestational diabetes, pre-eclampsia). Premature offspring or offspring with chromosomal abnormalities were excluded as we expected these to have a different aetiology. The exposure of interest was measured maternal vitamin D status (s-25OHD) in the blood/serum of the mother, cord, or offspring in the period leading up to pregnancy, during pregnancy, or shortly following birth, or exposure to vitamin D via supplementation in a randomised controlled setting. The outcome of interest was CAs in the offspring, including anomalies of the nervous system, circulatory system, respiratory system, digestive system, genital organs, urinary system, and musculoskeletal system, as well as malformations of the eye, ear, face and neck, cleft lip, and cleft palate. 

### 2.3. Information Sources

PubMed, Embase, and Cochrane Library were searched for relevant studies. The comprehensive literature search was performed in August 2019 (with no restriction on date) and again on 15 June 2020 (dates restricted to 2019–2020). 

### 2.4. Search

Search terms were identified by the authors of the present systematic review for the aspects of “fetal life” and “vitamin D”. All search terms were entered as free text as well as Medical Subject Headings (MeSH terms). The electronic search was limited to studies on humans by applying the filter “human” in the electronic databases. The search strategy was conducted by KCW after guidance from a librarian specialised in literature searches and was cross-checked by MNH (Table S1)

### 2.5. Study Selection

All identified studies were exported to Endnote and subsequently imported to the software programme Covidence, and duplicates were removed. The selection process was managed in the Covidence software by three reviewers (MNH, FT, and KCW). Initially, studies were assessed for inclusion based on title and abstract by independent votes of two reviewers. At this stage, studies were included based on relevant population and intervention, i.e., healthy pregnant women including their offspring and measured vitamin D or vitamin D supplementation in an RCT setting. Subsequently, full texts were retrieved, and studies were screened and included based on the outcome of independent votes of two reviewers. Disagreements during the selection process were resolved by discussion among the three reviewers (MNH, FT, and KCW). 

### 2.6. Data Collection

One author extracted relevant data from the included studies (KCW), and the extracted data were double-checked by either MNH or FT. The journal article, Supplementary Materials provided in the journal article, trial protocol/registration, and personal communication with study authors were used as sources to inform data extraction. We emailed the corresponding author of potentially relevant studies for clarification or to resolve other uncertainties. The deadline for a reply from the study authors was kept open during the conduction of the review. 

### 2.7. Data Items

Relevant study data included the following: study author, country, year of publication, study design, size of the study population, source of the study population (in- and exclusion criteria), baseline characteristics, assessment of exposure, trimester of exposure (if specified), confounding factors considered, outcomes and method of assessment, analysis methods, and key results, as well as reported conflicts of interest and trial registration. 

### 2.8. Summary Measures and Synthesis of Results

Results of the included RCTs were synthesised in a meta-analysis with random effect inverse variance analyses to generate pooled effect estimates expressed as relative risk and corresponding 95% confidence interval (CI). Heterogeneity was quantified using I^2^ statistics, with an I^2^ value greater than 50% considered as substantial heterogeneity.

Additional planned analyses, i.e., funnel plots, dose-response analyses, subgroup analysis by risk of bias, and types of anomalies, were deemed inappropriate due to a lack of studies.

All Analyses Were Performed in Revman Version 5.4 (Cochrane, London, UK). A meta-analysis of observational studies was planned but deemed limited due to the unavailability of eligible studies. Therefore, a narrative synthesis was conducted. In accordance with the guideline in the Risk of Bias In Non-Randomised Studies of Intervention (ROBINS-I) tool [14], we excluded studies that were assessed to have a critical risk of bias from the synthesis. 

### 2.9. Certainty Assessment (GRADE)

Certainty assessments in the effect estimates were evaluated using the Grading Recommendations Assessment, Development, and Evaluation (GRADE) method, with the four possible quality ratings: high, moderate, low, and very low. Downgrading was performed using the standard definition of risk of bias, inconsistency, indirectness, imprecision, and publication bias. The overall certainty in the evidence was based on the lowest quality of the primary outcome. 

### 2.10. Risk of Bias in Individual Studies

Risk of bias was assessed by two reviewers independently (MHN and KCW) by applying the Cochrane Risk of Bias 2 (RoB 2) tool [15] and the ROBINS-I tool [14] in RCTs and observational studies, respectively. Discrepancies were resolved through discussion. According to ROBINS-I [14], confounder domains and co-interventions should be specified at the protocol stage. Confounders were found by constructing and analysing a directed acyclic graph, including maternal age, body mass index (BMI), smoking, socio-economic status, ethnicity, parity, and alcohol consumption. Possible co-interventions that individuals might receive after the initiation of the exposure if interest could be multivitamin use or counselling from a trained dietician. This may be initiated if inadequate vitamin D status is detected during pregnancy. 

## 3. Results

**Figure 1:**
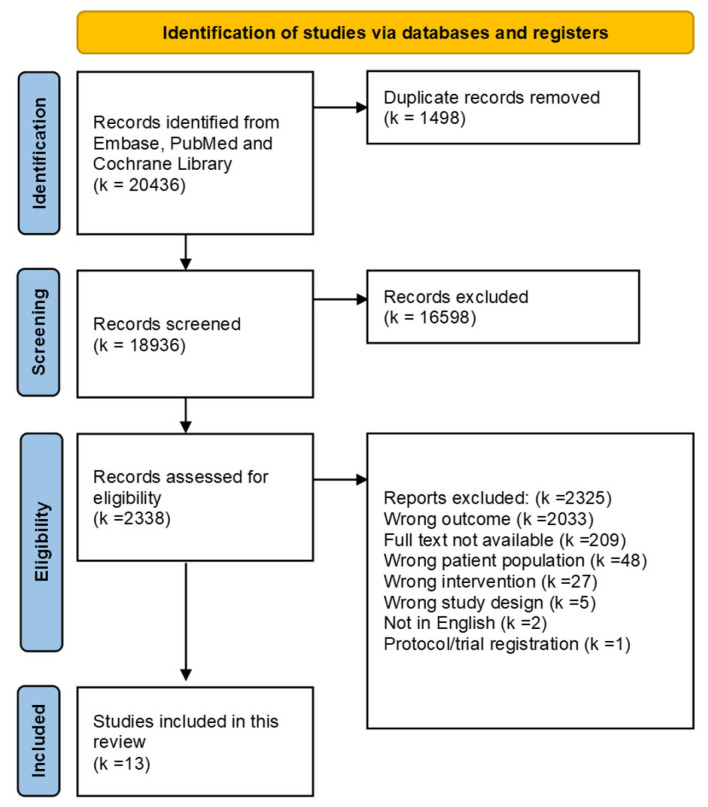
Flow chart.

The comprehensive literature search yielded a vast number of unique records (18,938 records). After the removal of duplicates, all records were screened by title and abstract, resulting in 2338 records that were retrieved and assessed in full. The full-text assessment led to the exclusion of 2325 records, and a total of 13 studies were included in the review (Figure 1). Detailed reasons for the exclusion of the 2325 studies can be presented upon request. The complete search strategy can be seen in Table S1.

### 3.1. Study Characteristics

Four RCTs [16,17,18,19] and nine observational studies [20,21,22,23,24,25,26,27,28] were included in the review. The RCTs were published between 2016 and 2018 and originated from the USA [18], Canada [19], the UK [17], and Denmark [16], and varied in study population size from 623 to 1298 included pregnant women. The intervention was supplementation with vitamin D ranging from a daily supplement of 800 IU to weekly supplements of 28,000 IU. The primary outcomes of the four included RCTs were persistent wheeze, bone health, asthma and recurrent wheeze, and infant growth. Roth et al., included congenital malformations as an outcome [19], while the remaining three RCTs included congenital malformations as a safety measure [16,17,18]. 

The observational studies were published between 2014 and 2018. Five of the nine studies were conducted in Turkey [22,24,25,26,28]. The remaining studies were from Spain [20], Egypt [27], Tunisia [23], and China [21] and varied from small case-control studies of 60 participants to cohort studies of 1953 participants. Vitamin D status (s-25OHD) was assessed in maternal blood during gestational weeks 11–25, except for one study that assessed vitamin D status in mothers and offspring shortly after birth [26]. One study did not specify when vitamin D status (s-25OHD) was assessed [27]. The primary outcome of the observational studies were malformations, NTDs, CHD, and congenital diaphragmatic hernia (see Table 1, Table 2 and Table S2).

### 3.2. Risk of Bias within Studies

Risk of bias was assessed for all included studies and presented separately for RCTs and observational studies (see Figure S1a,b).

The four included RCTs were all assessed to be at some concern of bias. The judgement was driven by domain 5, “Bias in selection of the reported result”, according to which, all studies were judged as having some concerns due to missing a pre-specified analysis plan. Chawes et al., 2016 [16] and Litonjua et al., 2016 [18] both had missing information concerning allocation sequence concealment and were judged to be at some concerns of risk of bias in domain 1, “Bias arising from the randomisation process”. See Figure S1a. 

All nine observational studies were assessed to have a serious or critical risk of bias, with the most problematic domain in ROBINS-I being domain 1 “Bias due to confounding”. Six observational studies did not adjust analyses for any confounders and were subsequently excluded from the synthesis in accordance with guidelines from ROBINS-I [14]. Three observational studies adjusted for some confounders and were rated at serious risk of bias [21,23,26]. See Figure S1b. 

### 3.3. Vitamin D and Congenital Anomalies

**Figure 2:**
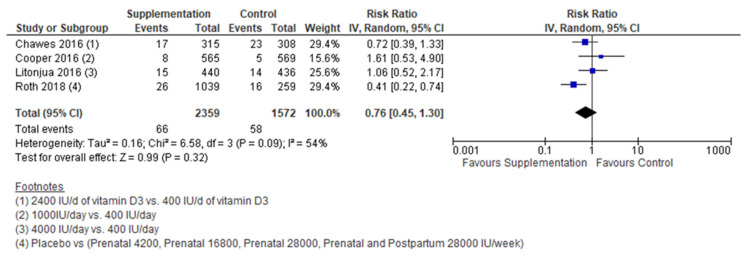
Meta-analysis of RCTs [16,17,18,19].

The meta-analysis of the four RCTs (*n* = 3931) that reported on CAs showed no difference in the risk of CAs between vitamin D supplementation and placebo (RR: 0.76, 95% CI: 0.45, 1.30) (Figure 2). There was substantial heterogeneity (I^2^ = 54%, *p* = 0.09), and a sensitivity analysis showed that by removing the study by Roth et al., 2018 [19], the heterogeneity was reduced to I^2^ = 0%. The certainty of the effect estimates was moderate due to imprecision (wide confidence intervals). We did not assess risk of publication bias due to the low number of included studies. We found discrepancies in the reporting of CAs in the Supplementary Materials and the clinical trial’s registration in the study by Litonjua et al., 2016 [18]. We extracted the data from the peer-reviewed Supplementary Materials. This did not substantially affect the overall effect estimate of the meta-analysis.

The three included observational studies investigated the association between vitamin D status and CHD, congenital malformations, and NTD, respectively [21,23,26] (see Table 3). Further, the studies differed in terms of the timing of s-25OHD assessment, from 11–25 weeks of gestation, shortly after birth or not reported, and the selection of confounding factors. Only one study found a significant association; Nasri et al., 2016 [23] found decreased concentrations of vitamin D to be associated with an increased risk of neural tube defects, with an adjusted odds ratio of 2.34 95% CI (1.07; 5.07) and a *p*-value 0.035. Of note, the cases and controls were matched by folate supplementation in the study. 

We did not conduct a GRADE assessment for the observational studies as we were not able to pool the estimates. 

## 4. Discussion

### 4.1. Summary of the Evidence

Maternal nutrition and supplement use during pregnancy are important for foetal development, and certain nutrients have been linked to the development of CAs, i.e., folic acid, for the prevention of NTDs [29]. This systematic review showed that, with overall moderate certainty in our findings, supplementation with vitamin D up to 4000 IU/day during pregnancy was not associated with differences in the occurrence of CA, though the RR in the meta-analysis pointed towards being in favour of supplementation. Although there were some variations in baseline characteristics, vitamin D dose, intervention duration, and outcome definition of the included RCTs, the evidence from the systematic review and meta-analysis was based on well-conducted and methodologically strong RCTs. A caveat to our results is that the included RCTs were not designed to answer our research question, and CAs were included in most of the RCTs as safety measures. CAs are rare events, particularly when considering the individual types of anomalies, and our meta-analysis may be underpowered. Further, the inclusion of participants in the RCTs was after the first trimester and, thereby, after the initial organ formation and what we consider to be the critical window of exposure. Due to limitations imposed by the scientific ethical committee, Cooper et al., 2016 only included women with concentrations of vitamin D between 25 and 100 nmol/L and thereby excluded women with low concentrations of vitamin D [17]. This may have prevented any beneficial effect materialising due to sufficient concentrations of vitamin D already at baseline for all participants [30]. In fact, secondary analyses completed by Litonjua and colleagues revealed that initial concentrations of vitamin D among the participants affected the overall effect of the intervention [31].

The null results on vitamin D supplementation during pregnancy on offspring risk of CAs were also reported by previous reviews by Bi et al., 2018 and by Liu et al., 2022 [32,33]. Bi et al., 2018 did not include results from Roth et al., 2018 [19]. Neither of the two systematic reviews included observational studies, nor were CAs the primary objective of the reviews [32,33].

One of the included observational studies showed that vitamin D deficiency (<30 nmol/L) in the mother was associated with an increased risk of NTD in the foetus [23]. A similar result was found in two other observational studies that examined NTDs as an outcome; however, they are here excluded due to critical risk of bias [22,28]. Sources of vitamin D, e.g., fatty fish, are part of a healthy diet, and maternal vitamin D status may also be considered an indicator of the general nutritional status of the mother during pregnancy. Possible interactions between nutrients, e.g., folic acid and vitamin D, may be hypothesised and should be tested for in future studies.

Regarding other CAs, two of the included observational studies showed no association between s-25OHD and CHD and malformations, respectively [21,26]. This is in accordance with findings from two studies with malformations as the outcome that we excluded due to their critical risk of bias in accordance with the ROBINS-I [20,24]. In contrast, the excluded study with a critical risk of bias by Mokhtar et al., 2018 showed that maternal vitamin D deficiency (s-25OHD < 25 nmol/L) was associated with an increased risk of CHD in offspring [27].

Another study we excluded due to a critical risk of bias found that maternal serum vitamin D was significantly lower in pregnancies complicated by congenital diaphragmatic hernias than in healthy pregnancies [25]. The lack of appropriate confounder control invalidates the trust in the associations found in the excluded studies.

### 4.2. Strengths and Limitations of the Included Studies

The four included RCTs had sufficient quality to be included, together reaching a total participant number of 3931. By GRADE assessment, the certainty reached a moderate level given the wide confidence intervals. None of the RCTs had CAs as their primary endpoint, which is probably related to the low prevalence of CAs, making a single RCT on CA as a primary outcome non-feasible. The onset of the intervention varied between gestation weeks 11 and 25, i.e., the second trimester of pregnancy, which is after the critical period of organ formation early in pregnancy [16,17,18,19]. Only one RCT excluded known foetal anomalies before randomisation [18].

Reporting adverse events may be performed very differently across studies [34]. This has implications for the validity of the current review, as we cannot be sure that all CAs were identified. Further, the method of detection may vary and introduce heterogeneity between the RCTs. CAs were an a priori safety measure; however, the method of measurement of CAs was not stated, which gives rise to concerns about underreporting of less obvious CAs. Based on these limitations, we are careful to make conclusions about the association between vitamin D and CAs from the current level I (RCT) evidence.

The three included observational studies were at serious risk of bias, primarily due to incomplete confounder control but also due to the selection of participants, e.g., two of the studies restricted the inclusion of pregnant women to those giving live births and measurement of vitamin D status was performed at different time points and with different methods of detection. Nasri et al., 2016 included pregnant women before elective termination [23]. Furthermore, the case-control match by folate supplementation was not specified in detail which could call into question the quality of matching of this important nutrient regarding NTD.

### 4.3. Strengths and Limitations of the Review

The comprehensive literature search is a strength of the present systematic review. We searched for and included studies in which the aim was to assess the association between vitamin D status during pregnancy and CA in the offspring and those that examined vitamin D supplementation during pregnancy and other outcomes with CAs as a safety measure or secondary outcome. We successfully captured both the former and the latter.

Publications based on the same study population were only included with one publication. One study did not report CA as an outcome; however, it stated that one child died with a CA. We were not able to obtain contact with the author to clarify if CAs were included as an outcome and thereby routinely reported for all study subjects or only for deaths [35]. This study was not included in the present systematic review. RCTs without any CAs may not have reported their findings and thus were not included in the present systematic review.

CA is a composite outcome covering potentially very different conditions, and a limitation of this review is the lack of possibility to conduct subgroup analyses on types of anomalies, severity, or dose–response relationships. We were furthermore not able to perform meta-regressions or subgroup analyses to test the cause of the substantial heterogeneity due to the scarcity of studies. Heterogeneity was most likely due to differences in populations, interventions, comparisons, and outcomes.

## 5. Conclusions

This review provides an overview of the results from the available literature on vitamin D status and CAs, and the results from the meta-analysis of RCTs suggested no association between vitamin D and CAs, with moderate certainty in our findings. However, few and small studies prevented us from providing a firm conclusion on the association. Unlike for many other and more frequent outcomes, the most feasible study design to investigate the present research question is the observational study design due to the low prevalence of CAs and the recruitment of participants, as well as that the initiation of supplementation often takes place after organogenesis. However, most current observational studies lacked sufficient control of confounders.

Future studies in this area should consider including pregnancies before termination of pregnancy to avoid selection bias, and optimally, women of reproductive age should be included prior to pregnancy. Additionally, future studies should be of sufficient size and include relevant confounders.

## Figures and Tables

**Table 1:** Characteristics of included RCTs.

Author, Year	Sample Size	Maternal Baseline Characteristics (*Age, Body Mass Index (BMI), Smoking, Socio-Economic Status (SES), Ethnicity, Parity, and Alcohol Consumption*)	Inclusion Criteria	Exclusion Criteria
Chawes, 2016 [16]	Intervention group: 295 Control group: 286	Age (years), mean (SD) 32.3 (4.3) Smoking *n* (%) 46 (8) SES, educational level, *n* (%) Low: 45 (8) Medium: 375 (65) High: 160 (27) Parity, *n* (%) Primiparity: 263 (45) Remaining baseline characteristics not reported	Healthy pregnant women	Gestational age > week 26, any endocrine, cardiovascular, or nephrological disorders; or vitamin D3 (cholecalciferol) intake more than 600 IU/d.
Cooper, 2016 [17]	Intervention group: 565 Control group: 569	Age (years), mean (SD) Intervention group: 30.5 (5.2) Control group: 30.5 (5.2) BMI (kg/m^2^), median (IQR) Intervention group: 24.7 (22.3–28.6) Control group: 25.7 (23.0–30.0) Smoking (yes), *n* (%) Intervention group: 44/533 (8%) Control group: 43/526 (8%) SES, educational attainment ≥A level Intervention group: 414/531 (78%) Control group: 393/522 (75%) Ethnicity, white ethnic origin, *n* (%) Intervention group: 499/531 (94%) Control group: 497/527 (94%) Parity, Nulliparous, *n* (%) Intervention group: 232/532 (44%) Control group: 230/524 (44%) Alcohol consumption not reported	Women older than 18 years, had a singleton pregnancy, had gestation of less than 17 weeks based on last menstrual period and ultrasound measurements, and were aiming to give birth at the local maternity hospital. Women with a serum 25-hydroxyvitamin D (25[OH]D) concentration of 25–100 nmol/L and serum calcium of less than 2.75 mmol/L.	Metabolic bone disease, renal stones, hyper parathyroidism, or hypercalciuria, diagnosed with cancer in the previous 10 years, unable to give informed consent or comply with the protocol, taking drugs known to interfere with foetal growth, foetal anomalies on ultrasonography, or taking more than 400 IU/day vitamin D supplementation.
Litonjua 2016 [18]	Intervention group: 440 Control group: 436	Age, mean (SD) Intervention group: 27.5 (5.5) Control group: 27.3 (5.6) BMI (kg/m^2^) not reported Smoking All non-smokers SES, educational status, *n* (%) Intervention group: <High school: 66 (15) High School or technical school: 123 (28) Some college: 108 (25) College graduate or graduate school: 143 (33) Control group: <High school: 42 (10) High School or technical school: 142 (33) Some college: 105 (24) College graduate or graduate school: 147 (34) Ethnicity Intervention group: Black: 190 (43) White Hispanic: 59 (13) White non-Hispanic: 114 (26) Other: 77 (18) Control group: Black: 190 (44) White Hispanic: 61 (14) White non-Hispanic: 116 (27) Other: 69 (16) Parity not reported Alcohol consumption not reported	Women between 18 and 39 years, with estimated gestational ages of 10 and 18 weeks; who had a history of asthma, eczema, or allergic rhinitis, or whose partner (biologic father of the child) had a history of asthma, eczema, or allergic rhinitis; who was a nonsmoker; and who was English or Spanish speaking, with intent to participate for 4 years (up to the third birthday of the child).	Not reported
Roth, 2018 [19]	Placebo group: 259 Prenatal 4200 group: 260 Prenatal 16,800 group: 259 Prenatal 28,000 group: 260 Prenatal and Postpartum 28,000 group: 260	Age, median (range) Placebo group: 23 (18–38) Prenatal 4200 group: 22.5 (18–40) Prenatal 16,800 group: 22 (18–35) Prenatal 28,000 group: 22 (18–38) Prenatal and Postpartum 28,000 group: 23 (18–38) Smoking not reported SES, Secondary school education complete or higher, *n* (%) Placebo group: 52 (20.1) Prenatal 4200 group: 70 (26.9) Prenatal 16,800 group: 51 (19.7) Prenatal 28,000 group: 58 (22.3) Prenatal and Postpartum 28,000 group: 55 (21.2) Ethnicity not reported Parity, median (range) Placebo group: 2 (0–6) Prenatal 4200 group: 2 (0–5) Prenatal 16,800 group: 2 (0–5) Prenatal 28,000 group: 2 (0–5) Prenatal and Postpartum 28,000 group: 2 (0–4) Alcohol consumption not reported	Women at 18 years or above, 17 to 24 completed weeks of gestation (i.e., 17 weeks +0 days to 24 weeks + 0 days, inclusive) based on recalled last menstrual period and/or ultrasound. Intends to reside in the trial catchment area (including Hazaribag, Azimpur, Lalbag, and Kamrangirchar) for at least 18 months. Provides written informed consent.	History of any medical condition or medications that may predispose to vitamin D sensitivity, altered vitamin D metabolism, and/or hypercalcemia, including active tuberculosis or current therapy for tuberculosis, sarcoidosis, history of renal/ureteral stones, parathyroid disease, renal or liver failure, or current use of anti-convulsants. High-risk pregnancy based on one or more of the following findings by point-of-care testing: - Severe anaemia: haemoglobin <70 g/L assessed by Hemocue - Moderate–severe proteinuria: ≥300 mg/dL (3+ or 4+) based on urine dipstick - Hypertension: ≥1 systolic blood pressure reading ≥140 mm Hg and/or ≥1 diastolic blood pressure reading ≥90 mm Hg in repeat measurements taken at least one minute apart High-risk pregnancy based on one or more of the following findings by maternal history and/or ultrasound: - Multiple gestation - Major congenital anomaly - Severe oligohydramnios Unwillingness to stop taking non-study vitamin D or calcium supplements or a multivitamin containing calcium and/or vitamin D. Currently prescribed vitamin D supplements as part of a physician’s treatment plan for vitamin D deficiency. Previous enrolment in the trial during a previous pregnancy.

**Table 2:** Characteristics of included observational studies.

Author, Year	Sample Size	Maternal Baseline Characteristics (*Age, Body Mass Index (BMI), Smoking, Socio-Economic Status (SES), Ethnicity, Parity, and Alcohol Consumption*)	Inclusion Criteria	Exclusion Criteria
Fernández-Alonso, 2012 [20]	*n* = 466	Maternal baseline characteristics not reported for those who were included in the second phase analysis	Pregnant women attending their first prenatal (week 11–14 of pregnancy) visit at the Torrecárdenas Hospital, Almería, Spain.	Women with an increased risk for intrauterine foetal growth restriction, specifically hereditary or acquired thrombophilias.
Zhou, 2014 [21]	*n* = 1923 (Group A: *n* = 364 Group B: *n* = 932 Group C: *n* = 627)	Age (years), mean (SD) Group A: 29.2 (3.5) Group B: 29.5 (3.6) Group C: 30.3 (3.9) BMI (kg/m^2^), mean (SD) Group A: 20.28 (2.52) Group B: 20.44 (2.51) Group C: 20.67 (2.64) Remaining baseline characteristics not reported	Pregnant women ≥18 years of age, recruited at the hospital. Included delivery methods were normal delivery, abortion, and induced labour	Women were excluded if they did not provide informed consent; had increased liver enzymes by a factor of two or more above upper normal limits; chronic disease and tumor; if the women presented with severe infections or trauma before 13 weeks of gestation, including 13 weeks; pregnant women accompanied by severe infections, trauma, or perioperatively. Before 13 weeks of gestation, including 13 weeks, pregnant women taking corticosteroids, drug abuse (including alcohol)
Daglar, 2014 [22]	*n* = 60 (Case group: *n* = 30 Control group: *n* = 30)	Age (years), mean (SD) Case group: 26.1 (5.4) Control group: 27.9 (5.3) BMI (kg/m^2^), mean (SD) Case group: 26.3 (5.5) Control group: 26.1 (5.2) Smoking (yes), *n* (%) Case group: 4 (13.3) Control group: 3 (10) Remaining baseline characteristics not reported	Women were recruited from a referral hospital for high-risk pregnancies. The patient profile of the hospital were low–middle income socio-economic groups	Women with a known history or evidence of rheumatological or adrenal diseases, hepatic or renal failure, gestational diabetes, hypertensive disorders, and previous history of childbirth with neural tube defect were excluded from the study
Nasri, 2016 [23]	*n* = 132 (Case group: *n* = 68 Control group: *n* = 64)	Age, >30 years, *n* (%) Case group: 41 (60) Control group: 41 (64) Parity ≥1, *n* (%) Case group: 37 (54) Control group: 54 (84) Remaining baseline characteristics not reported	Pregnant women were recruited from a unit receiving all referrals of women carrying a foetus with severe neural tube defect between January 2012 and December 2013. A healthy pregnant woman with normal ultrasonography and normal obstetric history was matched to every woman presenting with a foetus with neural tube defect by date/month of conception and use of folate supplementation.	Women with hypertension, cardiac disease, atherosclerosis.
Ates, 2016 [24]	*n* = 229	Age (years), mean (SD): 29.49 (4.879) BMI (kg/m2): 25.3 ± 4.5 Smoking (yes), n (%): 15 (6.6) SES, Education, *n* (%) 0–5 years: 75 (35.2) 6–8 years: 38 (17.8) =9 years: 100 (46.9) Parity Nulliparous, %: 35.5 Remaining baseline characteristics not reported	Pregnant women attending their first antenatal visit at an outpatient clinic.	Multiple pregnancies and women with a history of thyroid, parathyroid, or adrenal disease; hepatic or renal failure; metabolic bone diseases and those taking medications that might affect vitamin D metabolism.
Turkmen, 2017 [25]	Case group: 24 Control group: 53	Age, mean (SD) Case group: 26.4 ± 5.7 Control group: 27.0 ± 5.1 BMI (kg/m^2^) Case group: 26.3 ± 4.8 Control group: 26.3 ± 3.9 Smoking *n* (%) Case group: 3 (12.5) Control group: 5 (9.4) SES not reported Ethnicity not reported Parity unclearly reported Alcohol consumption: exclusion criteria	Pregnant women were recruited from the high-risk pregnancy and antenatal clinics	Patients with a known history or evidence of rheumatologic or adrenal disease, hepatic or renal failure, gestational diabetes, hypertensive disorders, drug abuse, alcohol consumption, steroid use, or vitamin D supplementation were excluded from the study.
Dilli, 2018 [26]	Case group: 108 Control group: 103	Age Case group: 27.4 ± 5.8 Control group: 27.3 ± 5.8 BMI (kg/m^2^) ≥25, *n* (%) Case group: 34 (31.5) Control group: 39 (37.9) Smoking n (%) Case group: 59 (54.6) Control: 54 (52.4) SES Case group: Low 58 (53.7) Medium 42 (38.8) High 8 (7.4) Control group: Low 57 (55.3) Medium 42 (40.7) High 4 (3.8) Ethnicity not reported Parity, Nulliparous, n (%) Case group: 45 (41.6) Control group: 47 (45.6) Alcohol consumption not reported	Cases born between 35–42 weeks of gestation and diagnosed with congenital heart disease within the first month of life at the tertiary neonatal intensive care unit between May 2013 and May 2015. Healthy controls were matched on gestational week, postnatal age, and sex.	Not reported
Mokhtar, 2018 [27]	Case group: 50 Control group: 50	Age, median (min-max) Case group: 28 (17–38) Control group: 28 (19–37) BMI not reported Smoking not reported SES, Educational level; *n* (%) Case group: High: 3 (6) Medium: 19 (38) Low: 28 (56) Control group: High: 6 (12) Medium: 20 (40) Low: 24 (48) Ethnicity not reported Parity not reported Alcohol consumption not reported	Mothers giving birth to term neonates diagnosed with a congenital heart disease within the first two weeks of life recruited from a tertiary neonatal intensive care unit. Recruitment took place at Zagazig University Children’s Hospital in Egypt between January 2016 and May 2018. Control mothers were age-comparable and gave birth to age and sex harmonised term neonates with congenital heart disease.	Mothers of neonates suffering from sepsis, congenital infection, genetic syndromes, multiple congenital malformations, and mothers with a history of certain diseases, drug intake, or who experienced an infection during pregnancy.
Sirinoglu, 2018 [28]	Case group: 79 Control group: 99	Age, mean (SD) Case group: 27.4 (6.03) Control group: 31.02 (6.07) BMI not reported Smoking not reported SES not reported Ethnicity not reported Parity not reported Alcohol consumption not reported	This case control study was conducted between January 2014 and April 2016 at a tertiary referral hospital. The control group were selected among gestational age-matched women who had a normal targeted ultrasound during the second trimester (during the 16th week of gestation)	Not reported

**Table 3:** Key findings of the observational studies (excluding the studies rated as having critical risk of bias, as recommended by ROBINS-I).

1st Author, Year, Country	Vitamin D Assessment (Timing)	Analysis Method	Confounding Factors	Outcome	Key Findings	Authors’ Conclusion
Dilli, 2018, Turkey [26]	Maternal and infant blood (<30 days of life)	Multivariate analysis	Maternal age, multivitamin use, maternal education, socio-economic levels, maternal chronic diseases, maternal homocysteine, zinc, folate levels (ng/mL), gender of the infant.	Congenital heart disease	Odds ratio not reported for vitamin D	The authors found no significant association between vitamin D and congenital heart disease.
Nasri, 2016, Tunisia [23]	Maternal blood (Vitamin D was assessed ≤20 weeks of gestation for 43% of the neural tube defect group and for 42% of the control group and after 20 weeks of gestation for the remaining women)	Multivariate analysis	Odds ratios adjusted for maternal age, season of blood draw, pregnancy duration, foetal weight, gravidity, parity, and consanguinity	Neural tube defect	Odds ratio 2.34 95% CI (1.07; 5.07) *p*-value 0.035	The authors found that s-25OHD < 30 nmol/L in the mother was associated with an increased risk of having a foetus with neural tube defect.
Zhou, 2014, China [21]	Maternal blood (16–20-weeks of gestation)	Logistic regression analysis	Odds ratios adjusted for maternal age, systolic/diastolic pressure, pre-pregnancy body mass index, and serum calcium	Malformations	Odds ratio 1.016 * 95% CI (0.984; 1.049) *p*-value 0.338	The authors found no significant difference in malformations between the three groups *.

* According to authors, vitamin D was categorized into three groups. The three groups were based on 25OHD concentrations: Group A (low concentrations, ≤20 ng/mL), Group B (medium concentrations, 21–29 ng/mL), and Group C (high concentrations, ≥30 ng/mL). However, only one OR was presented. The statistical approach is unclear.

## Data Availability

The data presented in this study are openly available in the respective studies included in the systematic review.

## Supplementary Materials

The following supporting information can be downloaded at: https://www.mdpi.com/article/10.3390/nu15092125/s1, Table S1: Search terms and strategy; Table S2: Study identification; Figure S1: (a) Risk of bias assessment of included RCTs according to Cochranes Risk of Bias tool 2; (b) Risk of bias assessment of included observational studies according to ROBINS-I.
