# Supplementary material for: Vitamin D Supplementation and Vitamin D Status during Pregnancy and the Risk of Congenital Anomalies—A Systematic Review and Meta-Analysis
Source: Nutrients. 2023 Apr 28;15(9):2125. doi: 10.3390/nu15092125 (PMC10181330; doi:10.3390/nu15092125)
Supplement: Supplementary file 1 [file nutrients-15-02125-s001.zip › nutrients-2312567-supplementary.pdf]

Table S1. Search terms and strategy.

**Aspect 1**		**Aspect 2**
Fetal life	AND	Vitamin D
fetal life OR Fetus OR foetus OR fetal OR foetal OR foetale OR Pregnant OR Pregnancy OR Neonate OR fetal programming OR periconception OR periconceptional OR periconceptionally OR preconception OR preconceptional OR preconceptionally OR Newborn OR Maternal OR Prenatal OR prenatally		vitamin D OR 25-hydroxyvitamin D OR 1,25-dihydroxy-vitamin D OR calcitriol OR ergocalciferol OR cholecalciferol OR vitamin D2 OR vitamin D3 OR 1-alpha hydroxyvitamin D3 OR 1-alpha-hydroxy-vitamin D3 OR 1-alpha hydroxy-vitamin D3 OR 1-alpha hydroxy calciferol OR 1-alpha-hydroxy-calciferol OR 1-alpha hydroxycalciferol OR 1,25 dihydroxyvitamin D3 OR 1,25-dihydroxy-vitamin D3 OR 1,25 dihydroxycholecalciferol OR 1,25-dihydroxycholecalciferol OR 25-hydroxy-vitamin D OR 25 hydroxy vitamin D OR 25-hydroxycholecalciferol OR 25 hydroxycholecalciferol OR 25 hydroxyvitamin D OR alfacalcidol OR calcidiol OR calcitriol OR calciferol OR ergocalciferol OR cholecalciferol OR calcifediol OR ultra violet rays OR UV rays OR sunlight OR ergocalciferols

Search terms used in the search are presented in the following table. The example is for PubMed database. All terms were searched for as free text as well as MeSH terms. Filters applied: Human.

Table S2. Study identification.

Author, Year, Country	Study design	Outcome (method of assessment)	Trial registration	Conflict of interest
Ates, 2016, Turkey [24]	Cohort	Congenital Malformations (not reported)	Not reported	The authors declare no conflicts of interest
Chawes, 2016, Denmark [16]	RCT	Congenital Malformations, any (not reported)	Clinicaltrials.gov NCT00856947	Dr Bisgaard reports receiving consulting fees from Chiesi.
Cooper, 2016, UK [17]	RCT	Congenital abnormalities (not reported)	ISRCTN 82927713. European Clinical Trials Database, EudraCT 2007–001716–23.	The authors declare that "This work was supported by grants from Arthritis Research UK, Medical Research Council (MRC), Bupa Foundation, National Institute for Health Research (NIHR) Southampton Biomedical Research Centre, University of Southampton and University Hospital Southampton NHS Foundation Trust, and NIHR Musculoskeletal Biomedical Research Unit, University of Oxford. IS and AP were funded by the MRC (programme code U105960371). The work leading to these results was

				supported by the European Union's Seventh Framework Programme (FP7/2007–2013), projects EarlyNutrition and ODIN under grant agreements numbers 289346 and 613977. We are extremely grateful to Merck GmbH for the kind provision of the Vigantoletten supplement". The authors further declare other interests including personal fees and grants.
Daglar, 2014, Turkey [22]	Controlled cross-sectional	Neural tube defects; meningocele, meningomyelocele, encephalocele, anencephaly and fetal acrania (Ultrasonographic examinations)	Not reported	The authors declare no conflicts of interest

Dilli, 2018, Turkey [26]	Case-control	Congenital heart disease (confirmed by echocardiography or catheterisation.)	Not reported	The authors declare no conflicts of interest
Fernández-Alonso, 2012, Spain [20]	Cross-sectional	Congenital Malformations (not reported)	The study protocol was approved by the Research Ethics Committee of the study hospital.	Not reported
Litonjua, 2016, USA [18]	RCT	Major fetal or congenital anomaly (not reported)	clinicaltrials.gov (NCT00920621)	The authors declare that; "All authors have completed and submitted the ICMJE Form for Disclosure of Potential Conflicts of Interest.<br><br>Dr Litonjua reported receiving personal fees from UpToDate Inc and Springer Humana Press. Dr

				McElrath reported receiving grants from the National Institutes of Health (NIH). Dr O'Connor reported receiving grants from the NIH. Dr Bacharier reported receiving grants from the NIH and National Heart, Lung, and Blood Institute (NHBLI), and personal fees from Aerocrine, GlaxoSmithKline, Genentech/Novartis, Merck, Schering, Cephalon, DBV Technologies, Teva, Boehringer Ingelheim, AstraZeneca,WebMD/ Medscape, Sanofi, and Vectura. Dr Zeiger reported receiving grants from the NHBLI, AstraZeneca,<br>Aerocrine, MedImmune, Genentech, Merck, and GlaxoSmithKline and personal fees from Genentech, Novartis, GlaxoSmithKline, and TEVA. Dr Hornsby reported receiving an NIH ancillary grant. Dr Hawrylowicz reported receiving an NIH ancillary grant, a fellowhip grant from Wellcome Trust Clinical Training Research Fellowship, grant G100758 from the Medical Research Council Centre,

				and grants from Asthma UK, the Lord Leonard and Lady Estelle Wolfson Foundation, and the Alpha 1 Foundation. No other disclosures were reported.<br><br>VDAART was supported by grant U01HL091528 from the NHLBI. Additional support was provided by grant U54TR001012 from the National Centers for Advancing Translational Sciences (NCATS) for participant visits at the Boston Medical Center.
Mokhtar, 2018, Egypt [27]	Case-control	Congenital heart defects (confirmed by the pediatric cardiologist using ECHO)	Not reported	The authors declare no conflicts of interest
Nasri, 2016, Tunisia [23]	Case-control study	Neural tube defects	Not reported	The authors declare no conflicts of interest

		(not reported)		
Zhou, 2014, China [21]	Cohort	Malformations including nervous system, circulatory system, digestive system, reproductive system, urinary system, musculoskeletal, chromosome disorder and others. (not reported)	Not reported	The authors declare no conflict of interest
Roth 2018, Canada [19]	RCT	Congenital anomalies (not reported)	ClinicalTrials.gov (NCT01924013)	The authors declare no conflicts of interest
Sirinoglu, 2018, Turkey [28]	Case-control	Fetal anomaly of neural tube origin (sonographically detected)	Not reported	The authors declare no conflicts of interest
Turkmen, 2017, Turkey [25]	Controlled cross-sectional	congenital diaphragmatic hernia	Not reported	The authors declare no conflicts of interest

		(ultrasonographic examinations)		

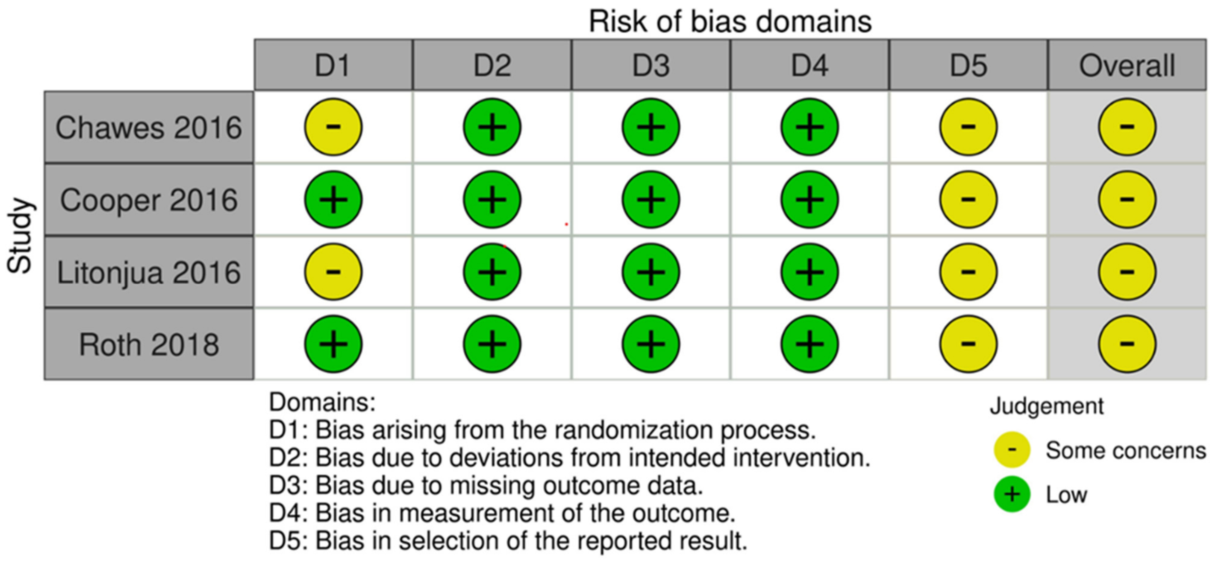

Figure S1a. Risk of bias assessment of included RCTs according to Cochranes Risk of Bias tool 2.

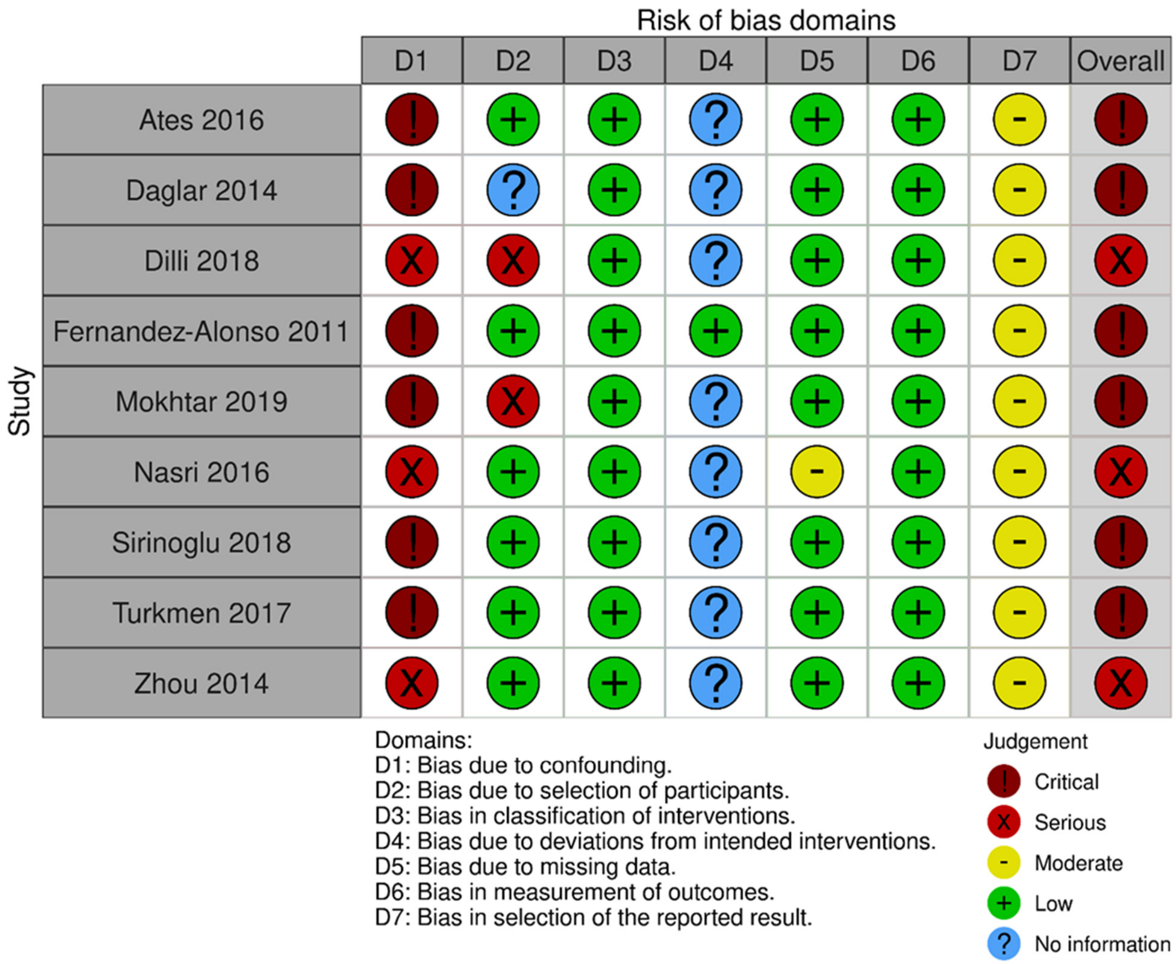

Figure S1b. Risk of bias assessment of included observational studies according to ROBINS-I.
